# Obesity in young-adult Nigerians: variations in prevalence determined by anthropometry and bioelectrical impedance analysis, and the development of % body fat prediction equations

**DOI:** 10.1186/1755-7682-5-22

**Published:** 2012-07-20

**Authors:** Chukwunonso ECC Ejike, Ifeoma I Ijeh

**Affiliations:** 1 Department of Biochemistry, College of Natural and Applied Sciences, Michael Okpara University of Agriculture, Umudike, PMB 7267, Umuahia, Abia State, Nigeria

**Keywords:** Anthropometry, Bioelectrical impedance analysis, Overweight/obesity, Young adults

## Abstract

**Background:**

Overweight/obesity is a growing global public health concern. The variations in the prevalence of overweight/obesity, defined by Body Mass Index (BMI), Waist Circumference (WC), Waist-to-Height Ratio (WHtR), Waist-to-Hip Ratio (WHpR) and Bioelectrical Impedance Analysis (BIA), were studied and a prediction equation for % body fat (%BF) developed.

**Methods:**

A total of 1584 subjects (56.4% males) were recruited for the study. Data on age, gender, height, weight, hip circumference and WC were collected from the subjects using standard protocols. BMI, WHtR and WHpR were derived using standard equations. %BF was measured using a BIA device (Omron BF-400). Appropriate statistical tools were used for the data analysis.

**Results:**

The prevalence of overweight/obesity in the population was 28.4% (36.3% for males; 22.6% for females) (BIA); 20.7% (17.5% for males; 24.8% for females) (BMI); 7.5% (1.3% for males; 16.1% for females) (WC); 2.9% (4.3% for males; 1.2% for females) (WHpR); and 15.4% (14.8% for males; 16.2% females) (WHtR). Taking BIA as the reference point, WC misclassified overweight/obesity the most for males (35%), while for the females, WHpR misclassified both disorders the most (21.4%). Correlation studies showed that only BMI correlated significantly, albeit weakly, with %BF among the males, whereas all the anthropometric measures, but WHpR correlated significantly with % body fat in females. Two prediction equations for %BF were generated, and %BF predicted with the two equations correlated significantly (*P* < 0.001) with that measured by BIA.

**Conclusion:**

The prevalence of overweight/obesity in this population vary widely depending on the definition used. The developed prediction equations could be useful in resource-poor settings, but require validation.

## Background

The prevalence of obesity has increased tremendously globally in both adults and children. In fact the World Health Organization (WHO) estimates that at least 1 billion people are overweight, and three hundred million of these are obese [1]. Overweight and obesity prevalence are reported to be increasing in developing countries, and mean Body Mass Index (BMI) levels are highest in middle income countries [2]. In Nigeria, a 2008 WHO report puts the prevalence of overweight and obesity at 26.8% and 6.5% respectively [3].

Young adults are reportedly prone to obesity in the transition from childhood/adolescence to adulthood [4,5]. The periods of strong oscillation and transition of body adiposity which occur in childhood and adolescence are thought to be the critical stages for the development of obesity [6]. In fact from age six, approximately half of obese children become obese adults, while only one tenth of non-obese children grow to be obese adults [7]. The prevalence of obesity in semi-urban dwelling adolescents in Nigeria is as high as 13.2% [8] and this does not bode well for any developing country, especially since obesity tracks into adult life with its co-morbid conditions [9] and affects productivity negatively.

The rising prevalence of obesity warrants the need for accurate methods of assessing adiposity. There are currently, however, many measures of obesity (anthropometric and otherwise). BMI despite its limitations, has become the most common indicator of overweight and obesity. It is used to reflect body fat, however, waist circumference (WC), waist-to-hip ratio (WHpR) and waist-to-height ratio (WHtR) are used as surrogates for visceral adiposity and predict obesity-related health risks better than BMI [10,11]. Accurate methods of estimating adiposity include underwater weighing, dual-energy X-ray absorptiometry (DXA), total body water, total body electrical conductivity/bioelectrical impedance analysis (BIA), total body potassium and computed tomography. The complexity and cost of these methods however limit their wide applicability in epidemiological studies [12]. Interestingly, non-invasive devices for the direct estimation of visceral fat area by BIA have been developed. In fact, a multi-frequency BIA device has been shown recently to be useful in assessing fat mass in healthy adults [13] and can be used in clinical and epidemiological research.

Given the dearth of information on overweight and obesity in young adults in Nigeria, and the palpable impact of the disorders on the health systems and economy of any nation, this study was designed to fill that vacuum by assessing the prevalence of overweight and obesity in an undergraduate population in Nigeria, using different anthropometric methods and the BIA method. Furthermore, given the cost and technicalities involved in the use of BIA in epidemiological studies, especially in resource-poor countries (typically in sub-Saharan Africa), this study also attempts to develop a % body fat prediction equation from easily assessable parameters.

## Subjects and methods

### Subjects

Participants in this cross-sectional study were undergraduate students of the Michael Okpara University of Agriculture, Umudike, Abia State, Nigeria (age range: 18–29 years) who were randomly recruited. Only those who gave an informed verbal consent, and who had no overt sign of ill-health nor reported present use of therapeutic medication or ‘substance’ use, were allowed to participate in the study. Pregnant women were also excluded. Data from 1584 subjects (56.4% males) were included in the analyses for this study.

## Methods

Self-reported age at last birthday was recorded per subject. Heights of the subjects were measured with an inelastic tape fastened to a vertical rod, to the nearest 0.1 cm, with the subject standing on bare feet. For weight measurements, the subjects were required to be on bare feet and wearing light clothing, while an electronic scale was used. Weight measurements were made to the nearest 0.1 kg. The waist circumference (WC) and hip circumference (HC) of each participant was measured using a non-elastic measuring tape, to the nearest 0.1 cm. WC was measured midway between the lowest rib and the superior border of the iliac crest at the end of normal expiration while HC was measured at the widest circumference over the buttocks. From these measurements WHpR was calculated as WC/HC; WHtR was calculated as WC/Height; and BMI was calculated as Weight (Kg)/[Height (m)^2^].

Body fat % was measured with a BIA device (Omron BF-400, Omron Healthcare Europe BV, Hoofddorp, The Netherlands) following the manufacturer’s instructions. The device sends an extremely weak electrical current of 50 kHz and less than 500 μA through the subject’s body and combines the electrical resistance with the distance of electricity conducted and the pre-entered particulars of the subject (age, sex, weight and height) to give the body fat %. The in-built formula used by the device was not disclosed by the manufacturer.

### Definitions

Overweight and obesity were defined by multiple definitions viz: (1) BMI ≥ 25 but < 30 and BMI ≥ 30 respectively [14]; (2) WC ≥ 80 for women and ≥ 94 for men [15]; (3) WHpR ≥ 1 [16]; (4) WHtR ≥ 0.5 [17] and (5) % body fat ≥ 32.0 (overweight) and ≥ 37.1 (obese) in black females and ≥ 21.7 (overweight) and ≥ 28.3 (obese) in black males [18].

### Statistical analysis

Descriptive statistics were carried out on the data generated. Continuous data are reported as means ± standard deviations, while categorical data are presented as percentages. Differences between means were separated by One Way ANOVA for continuous variables and by Chi Square test for categorical variables. Multiple regression analysis was used to test the association between % body fat on the one hand and BMI and other relevant variables on the other hand, and thus generate a prediction equation for % body fat in 50% of the subjects. For that analysis, gender was coded as 0 for females and 1 for males (as has been used by earlier authors). The correlation between the measured % body fat values and those obtained by the developed prediction equations were assessed (in the other 50% of the population) using the Pearson’s Product Moment Correlation Coefficients. The significant threshold was fixed at *P* < 0.05. Data analyses were done using SPSS version 18.0 (SPSS Inc. Chicago, IL). The results are presented in Tables.

## Results

The mean age of the participants was 21.8 ± 2.1 years, and males were significantly (*P* <0.05) younger than the females. All the obesity-related indices, except HC and WHtR, were significantly (*P* <0.05) different between the sexes (Table 1).

**Table 1:** Age and obesity-related clinical characteristics of the subjects

	**Male (893)**	**Female (691)**	*** P***	**Total (1584)**
Age (Yrs)	22.1 ± 2.1	21.5 ± 2.2	<0.001	21.8 ± 2.1
Height (cm)	171.8 ± 11.4	162.1 ± 6.4	<0.001	167.6 ± 10.7
Weight (kg)	66.7 ± 9.4	61.0 ± 9.1	<0.001	64.2 ± 9.7
Waist Circumference (cm)	77.3 ± 7.6	73.5 ± 7.8	<0.001	75.7 ± 7.9
Hip Circumference (cm)	89.9 ± 8.4	90.1 ± 8.7	0.566	90.0 ± 8.5
Body Mass Index (kg/m^2^)	22.4 ± 2.9	23.1 ± 3.3	<0.001	22.7 ± 3.1
Waist-to-Hip Ratio	0.86 ± 0.07	0.82 ± 0.07	<0.001	0.84 ± 0.07
Waist-to-Height Ratio	0.45 ± 0.04	0.45 ± 0.04	0.229	0.45 ± 0.04
% Fat Mass (%)	19.0 ± 6.5	28.2 ± 5.7	<0.001	23.1 ± 7.7

Based on BIA-determined body fat percentages, more males had more body fat than was ideal (36.3% as against 22.6% for females). Conversely, more females were overweight and/or obese based on BMI criteria. A total of 4.9% (3.9% for males and 6.1% for females) were thin; whereas 1.3% (0.4% for males and 2.5% for females) were obese. The prevalence of thinness, overweight and obesity were however similar (*P* >0.05) between the sexes. Significantly (*P* <0.05) more females had excessive waist circumference, relative to males. Males had a higher [albeit insignificantly so (*P* >0.05)] prevalence of elevated WHpR compared to females. Conversely, more females were found to have WHtR above the cut-off point, though this difference was not statistically significant (*P* >0.05) (Table 2).

**Table 2:** Prevalence of abnormal body composition in the population, defined by different criteria

	**Males (893)**	**Females (691)**	***P***	**All (1584)**
Thin (%)	18.7	28.1	0.133	22.8
Overweight (%)	26.0	15.5	0.077	19.5
Obese (%)	10.3	7.1	0.447	8.9
Thin (%)	3.9	6.1	0.527	4.9
Overweight (%)	17.1	22.3	0.205	19.4
Obese (%)	0.4	2.5	0.082	1.3
Overweight/Obese (%)	1.3	16.1	<0.001	7.5
Overweight/Obese (%)	4.3	1.2	0.174	2.9
Overweight/Obese (%)	14.8	16.2	0.845	15.4

*P* values are for Chi-square tests between the sexes.

The percentage discrepancy between overweight/obesity diagnosed by BIA, on the one hand, and the anthropometric determinants, on the other hand, are shown in Table 3. The proportion of overweight/obese males (by BIA) missed out was highest with the WC classification (35%). For the females, the WHpR missed out more overweight/obese subjects (21.4%) relative to the other anthropometric classifications. Overall, BMI had the least percentage discrepancy from the BIA data.

**Table 3:** Discrepancy between overweight/obesity diagnosed by BIA and by the other anthropometric methods

		% Discrepancy			
		**BMI**	**WC**	**WHpR**	**WHtR**
% Body Fat	Male	−18.8	−35.0	−32.0	−21.5
	Female	2.2	−6.6	−21.4	−6.4

% Discrepancy is calculated as % of population diagnosed as overweight/obese by anthropometric definitions – % of population diagnosed as overweight/obese by BIA.

Correlation studies showed that only BMI correlated significantly, albeit weakly, with % body fat among the males, whereas all the anthropometric measures, but WHpR, correlated significantly with % body fat in females (Table 4).

**Table 4:** Correlation between % body fat and other anthropometric measures of obesity

		**BMI**	**WC**	**WHpR**	**WHtR**
% Body Fat	Male r (*P*)	+0.094 (0.004)	−0.010 (0.758)	−0.008 (0.858)	+0.002 (0.946)
	Female r (*P*)	+0.834 (<0.001)	+0.422 (<0.001)	+0.067 (0.078)	+0.362 (<0.001)

From the results of the regression analysis, two prediction equations for % body fat were generated, viz:

1. 19.524 + 0.174(BMI) + 0.110(Age) – 0.440(Sex); and

2. 8.870 + 0.186(WC) + 0.158(BMI) + 0.098(Age) – 0.488(Sex).

Table 5 shows that % body fat predicted with the two equations correlated significantly (*P* < 0.001) with that measured by BIA.

**Table 5:** Correlation between measured and predicted % body fat values

		Predicted % Body Fat	
		**Equation 1**	**Equation 2**
Measured % Body Fat	r	+0.203	+0.208
	*P*	<0.001	<0.001

## Discussion

Obesity is a metabolic disorder that is typified by an increase in body fat and body weight. It is however the degree of increase in body fatness, not excess weight that is the predictor of health risk, yet body fat is not easily measured [19]. The measurement of BMI as a universal criterion of overweight (≥25) and obesity (≥30) is recommended by the WHO [16]. The primary assumption of BMI guidelines is that BMI is linked with body fatness and its associated morbidity and mortality [20]. However, it is possible for an individual to be obese yet lacking in the metabolic markers of adverse health risk. In fact, BMI-metabolic risk sub-phenotypes have been reported, and the proportion of the adult Nigerian population who are “metabolically obese normal weight” (MONW) and/or “metabolically healthy obese” (MHO) is arguably high [21,22]. These raise questions about the validity of BMI as a universal indicator of excess body fat. Other measures of obesity such as WC [23], WHpR [24] and WHtR [17] which define abdominal fat distribution have been suggested to be superior to BMI in predicting CVD risk [25]. These indices are not without limitations and criticisms. The direct measurement of body fat is now possible [13] and healthy % body fat ranges have been suggested [18]. This study therefore investigated the prevalence of overweight/obesity in a young-adult population in Nigeria.

### Prevalence of obesity determined by BMI standards

The 19.4% (17.1% for males and 22.3% for females) prevalence of overweight and the 1.3% (0.4% for males and 2.5% for females) prevalence of obesity reported in this study are lower than the 2008 WHO report on Nigeria which gave 26.8% and 6.5% for overweight and obesity, respectively [3] and the 20% (overweight) and 5% (obesity) reported in two villages in South-Western Nigeria [26]. Our figures are also lower those recently reported in a city in Northern Nigeria where overweight and obesity prevalence were as high as 53.3% and 21% respectively [27]. Obesity figures from other African countries are also higher than those reported here. In Ghana, obesity prevalence is reported to be 13.6% [28] while the figure is 18% for the Republic of Benin [29]. An obesity prevalence of 19.2% was recently reported in Dar es Salam, Tanzania [30]. Beyond Africa, in Portugal, the figures are as high as 36.4% (overweight) and 15.1% (obesity) [31] while in Spain they are 34.2% (overweight) and 13.6% (obesity) [32]. In the US, the prevalence of obesity has risen from 22.9% in the late 1980s and early 1990s to 30.5% between 1999 and 2000 [33]. However, a study of young adults (18–27 years old) in Hong Kong reported a prevalence of 13.2% and 26.7% in males and females respectively, for overweight/obesity [34]. Clearly the figures presented in this report are one of the smallest in the literature. This may be due to the younger age group of the subjects in this study, especially as obesity is known to increase with age [35]. Furthermore, we studied an undergraduate population who due to a high literacy level may be aware of the disadvantages of excess weight and may be working hard at maintaining a healthy weight. This is even more so in females who have more “body shape dissatisfaction” [34]. It is important to note, however, that the rigors of undergraduate work in Nigeria is often stressful on the students, the majority of whom are often poor. This, coupled with the active lifestyle of an average undergraduate student, may be responsible (at least in part) for the low prevalence of obesity in this population. Though overweight and obesity are known to affect more females than males, and more females than males were so-affected in this population, the differences between the sexes for both disorders were not statistically significant (*P* < 0.05). The low figures reported in this study for those who have exceeded the recommended threshold for a healthy body habitus are encouraging and efforts need to be intensified to encourage these (and other) young adults to strive for, and maintain, healthy weights as they grow into full adulthood.

### Differences between obesity diagnosed by BIA and by the anthropometric indices

One of the major findings of this study is the wide discrepancy in the prevalence of overweight/obesity as diagnosed by BIA and by anthropometric indices. The variations in prevalence exist even between different anthropometric definitions of overweight/obesity. Overweight/obesity diagnosed by BMI standards for example missed out as much as 18.8% of the male population who were detected by BIA. Conversely, 2.2% of the females diagnosed as overweight/obese had normal % body fat as determined by BIA. This poses a lot of health concern in affected individuals and at the population level as the opportunity to intervene and reduce the health risks associated with obesity is lost in such cases of misclassifications. Overall, BMI had the least % discrepancy. This is contrary to the reports of Kennedy *et al.*[36] who reported that BMI had the poorest ability to predict true adiposity.

Though anthropometry is accepted as a universal criterion for the diagnosis of overweight/obesity, its ability to define adiposity status has been constantly queried [37]. Previous reports have shown that BMI is not accurate in predicting adiposity status in subjects of all weight classifications [38] even when adiposity is determined by BIA [39]. Furthermore, a significant number of people with a BMI < 30 kg/m^2^ were actually obese when classified by BIA [40]. The problem with BMI in relation to adiposity is largely its inability to identify differences in body composition and body fat distribution.

Waist circumference has been proposed to be a better measure of obesity, relative to BMI [41]. However, WC captures abdominal obesity but does not reflect fat mass that may be distributed in non-abdominal tissues because it does not take height into cognizance. This is a major demerit of using the WC and may be responsible for the observed wide discrepancy in the prevalence of obesity diagnosed by BIA and by WC.It may also be responsible for the absence of a significant correlation between WC and % body fat in the male subjects (though the correlation was modest and significant in the females). The sex-specific differences may be a pointer to differences in sites of fat storage as the correlations seem to suggest that probably the bulk of the fatness in the females was around the waist. The higher prevalence of overweight/obesity (defined by WC) among the females appears to lend credence to this view.

The WHtR apparently corrects for height and is known to identify individuals at risk of health consequences of excess weight. It is believed to be better than WC as a global clinical screening tool [42]. The index is reported to be an excellent predictor of such adiposity-related disorders as the metabolic syndrome [43]. Yet it misdiagnosed as much as 21.5% of males and 6.4% of females, who were obese by BIA standards. A limitation of the WHtR may be that the WC measurement assesses only visceral adiposity such that dividing it by the subject’s height wrongly distributes the fatness localized around the abdomen to the entire body. This may explain the discordance between WHtR and % body fat in the diagnosis of obesity.

The WHpR is thought to be better than BMI, WC and WHtR as a measure of adiposity because of the distinct physiologic characteristics of different fat depots. Visceral fat has a lower threshold for lipolysis relative to subcutaneous fat, and free-fatty acids released by lipolysis have direct access to the liver. In this way, their metabolic consequences could be accentuated [10]. Expansion of visceral fat is also reported to alter the production of bioactive peptides with numerous local and systemic effects [44]. Conversely, subcutaneous fat appears to act as a sink for free-fatty acids [10], and higher subcutaneous fat has been associated with metabolic benefits in older persons [45]. The WHpR therefore serves as a superior composite factor subsuming the harmful effects of visceral fat and the beneficial qualities of subcutaneous fat [10]. However, % body fat estimates the body fat content of the entire body and not just those localized around the waist and hip. This may explain the degree of discordance observed between % body fat and WHpR.

The high degree of discordance between overweight/obesity diagnosed by BIA and that determined by the studied anthropometric variables suggests that the anthropometric variables under-diagnose obesity in the studied population. The implications of this on public health are enormous especially as many people thought to be of “normal weight” by anthropometric standards may have more % body fat than is required. This observation may account for the MONW phenotype observed in populations in Nigeria [21,22].

### Prediction of % body fat from other relevant factors

The anthropometric indices – BMI, WC, WHtR, WHpR – are known surrogate markers of excess adiposity, however, it is more ideal to use actual measures of fatness rather than surrogates [20]. BIA is considered by many as a more accurate method for estimating % body fat [40,46]. The development of machines that use the BIA method has made the assessment of % body fat easier [13], yet the machines are relatively costly especially in rural communities and often require skilled manipulation. The development of prediction equations that use indices derived from easy-to-use and affordable techniques is therefore desirable. The equations we developed use BMI, age and sex in one case, and WC added in another case. The other studied variables did not enter the regression models. Many cross-sectional and longitudinal studies show that % body fat increases with age in both males and females [47,48] and that underscores the importance of age in any prediction equation for % body fat. Similarly, % body fat was significantly higher in the females relative to the males in this study, a result that is corroborated by earlier studies [18,20] and which justifies the inclusion of a gender factor in the equation. The inclusion of BMI and WC is justified since both are widely used measures of adiposity and contain variables found in the anthropometric indices excluded by our models. Though BMI could be transformed to improve the linearity of its relationship with % body fat, Zhu *et al*. [18] report an identical coefficient of determination for regressions modeled by BMI, BMI^2^ or 1/BMI, thus making our use of BMI acceptable. The very significant correlation between the % body fat generated using both equations and the measured % body fat lends weight to the usefulness of the equations as alternatives in places where the BIA equipment is unavailable. Our equations form a preliminary model that should be validated by further studies.

### Limitations and strengths

This study may be limited by our choice of sample. Our subjects necessarily were a convenient sample and may not represent that age bracket in the general population. Furthermore, we did not include older adults in this study, even though morbidity from adiposity-related disorders is more prevalent in older adults. Our sample size is also not large enough to allow for robust statistical deductions to be made without equivocation. However, in the light of our peculiar clime, it is enough starting point for future studies. The assumption that % body fat is an improved phenotypic indicator of morbidity and mortality over BMI, WC etc. is still debatable and more studies are needed to clarify the issues surrounding body composition. This is compounded by our inability to measure biochemical markers of adiposity. The discordance reported in the prevalence of overweight/obesity in the studied population should therefore be interpreted with caution. Though the BIA technique is known to be problematic in cases of morbid obesity where an increased amount of total body water and extracellular water could lead to an under-estimation of % body fat, we feel that our results may not have been affected by that, since the prevalence of obesity in this population was low and we did not recruit morbidly obese subjects.

The strength of our study lies in its novelty and its recruitment of subjects who were considerably young (though adults) and apparently healthy. This allowed for the exclusion of other chronic (and communicable) diseases that may have confounding effects on the analyses. The development of % body fat prediction equations would, no doubts, positively improve epidemiologic studies and health care monitoring and delivery especially in resource-poor settings.

## Conclusion

The prevalence of overweight/obesity in this young adult Nigerian population varied (depending on the definition of obesity) from 2.9% to 28.4%. Generally females were more overweight/obese than males. The % discrepancy between diagnosis made using BIA and the anthropometric variables were widest when WC was used in males and when WHpR was used in females. Two % body fat prediction equations were developed and each correlated significantly with the measured values. There is a need to harmonize what each measure of adiposity should be used for (diagnostically). Further studies are also warranted to investigate human body compositions further and develop robust equations for predicting % body fat from simple anthropometric variables.

## Competing interests

The authors declare that they have no real or potential conflict of interests.

## Author contributions

CECCE was responsible for the conception and design of the study, supervision of data collection, data analysis and writing of the manuscript. III contributed to the design of the study, supervised data collection and vetted the draft manuscript for intellectual content. Both authors read and approved the final version of the manuscript.
